# Distal Kaplan fibers and anterolateral ligament injuries are associated with greater intra-articular internal tibial rotation in ACL-deficient knees based on magnetic resonance imaging

**DOI:** 10.1186/s40634-023-00682-0

**Published:** 2023-11-09

**Authors:** Luca Farinelli, Amit Meena, Bertrand Sonnery-Cottet, Thais Dutra Vieira, Charles Pioger, Sachin Tapasvi, Elisabeth Abermann, Christian Hoser, Christian Fink

**Affiliations:** 1 Clinical Orthopaedics, Department of Clinical and Molecular Sciences, Università Politecnica Delle Marche, Ancona, Italy; 2 Gelenkpunkt - Sports and Joint Surgery, Olympiastraße 39, Innsbruck, 6020 Austria; 3 Research Unit for Orthopaedic Sports Medicine and Injury Prevention (OSMI), Private University for Health Sciences, Medical Informatics and Technology, Innsbruck, Austria; 4 Centre Orthopedique Santy, FIFA Medical Centre of Excellence, Hôpital Mermoz, Groupe Ramsay, Lyon, France; 5 Department of Orthopaedic Surgery, Centre Hospitalier de Versailles, 177, Rue de Versailles, Le Chesnay, 78157 France; 6 The Orthopaedic Speciality Clinic, Pune, India

**Keywords:** Anterolateral complex, Kaplan fibers injuries, Anterolateral ligament, ACL, Internal tibial rotation, MRI

## Abstract

**Purpose:**

The purpose of the present study was to assess the internal rotation of the tibia on Magnetic Resonance Imaging (MRI) in a series of consecutive athletes with Anterior cruciate Ligament (ACL) tears.

**Methods:**

Retrospective analysis of prospectively collected data was performed to include all consecutive patients who had undergone primary ACL reconstruction between January 2022 and June 2022. The angle between surgical epicondylar axes (SEA) of the knee and posterior tibial condyles (PTC) was measured. A negative value was defined as internal torsion. KFs and ALL injuries were reported. Analysis of covariance (ANCOVA) was performed to examine the independent associations between SEA-PTC angle and injuries of KFs and ALL adjusted for physical variables (age, gender and body mass index [BMI]). Statistical significance was set at a *p*-value of < 0.05.

**Results:**

A total of 83 eligible patients were included. The result of multiple linear regression analysis showed that internal tibial rotation was associated with KFs and ALL injuries. The estimated average of SEA-PTC angle in relation to ALL injuries controlling the other variables was -5.49 [95%CI -6.79 – (-4.18)] versus -2.99 [95%CI -4.55 – (-1.44)] without ALL injuries. On the other hand, the estimated average of SEA-PTC angle in relation to KFs lesions controlling the other variables was -5.73 [95%CI -7.04 – (-4.43)] versus -2.75 [95%CI -4.31 – (-1.18)] without KFs injuries.

**Conclusions:**

KFs and ALL injuries were associated with an increased intra-articular internal tibial rotation in ACL-deficient knees. The measurement of femorotibial rotation on axial MRI could be useful to detect indirect signs of anterolateral complex (ALC) injuries.

## Introduction

Anterior cruciate ligament (ACL) rupture represents a serious injury for athletes [10]. It could be associated to meniscal, cartilage and collateral ligament lesions and represent a common cause of rotatory knee instability [9, 13, 33]. The ACL provides restraint to anterior translation and internal rotation of the tibia relative to the femur [16, 38, 50]. Vassalou et al. reported that patients with acute and chronic ACL tears had an internal tibial rotation measurements of 10.7 and 11 degrees respectively [50]. Moreover, Mitchell et al. found a significant increase in internal tibial rotation in ACL-deficient knees compared to intact knees in the adolescent population [38]. Hong et al. reported that aged patients with ACL tears exhibited significantly greater tibial internal rotation compared to younger patients (5.6° vs 4.2°) hypothesizing that older patients might have a higher incidence of associated injuries [26]. With the spread of Magnetic Resonance Imaging (MRI), several findings have been proposed to indicate ACL tears and static signs of anterolateral rotatory instability such as anterior tibial translation [6, 36], bone kissing contusions [47], and internal tibia rotation [38, 50]. Associated injuries that could lead to internal rotation of the tibia in the setting of ACL injury need to be clarified. Previous controlled laboratory studies reported that injuries of the anterolateral complex (ALC) such as Kaplan Fibers (KFs) and anterolateral ligament (ALL) injuries result in increased internal rotation of the tibia in ACL-deficient knees [18, 31, 42, 45].

The purpose of the present study was to assess the internal rotation of the tibia on MRI images of a series of consecutive athletes with ACL tears. We hypothesized that KFs and ALL injuries were associated with greater internal rotation on pre-operative MRI.

## Material and methods

**Fig. 1:**
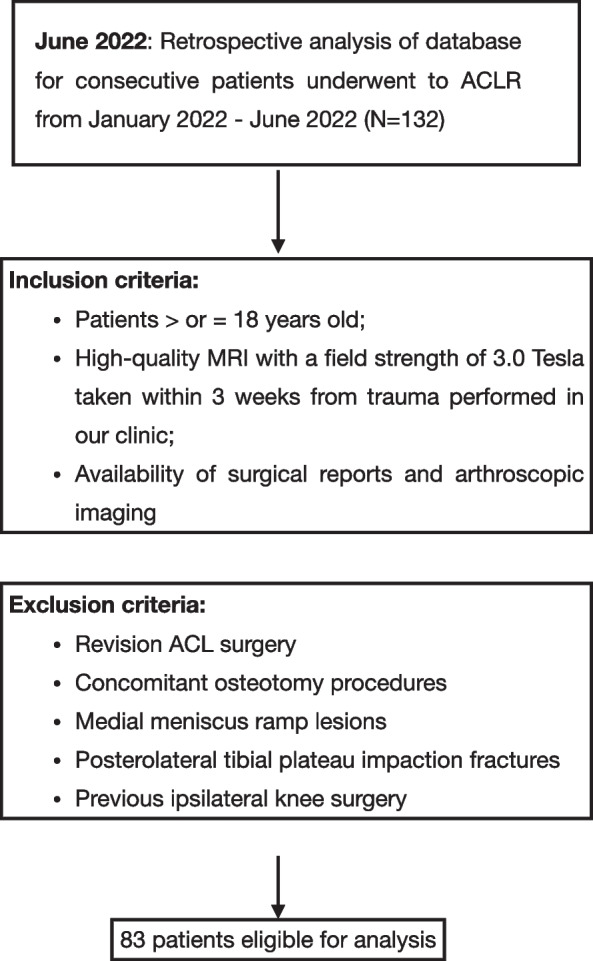
Flow chart of the study. MRI: magnetic resonance imaging; ACL: anterior cruciate ligament

The present retrospective study was conducted following the Declaration of Helsinki Ethical Principles and Good Clinical Practices and was approved by the ethical committee of the Medical University of Innsbruck (AN2015-0050 346/4.28). A retrospective analysis of prospectively collected data from the database of a specialized joint surgery clinic was conducted. All consecutive patients who underwent arthroscopic primary ACL reconstruction (ACLR) between January 2022 and June 2022 were considered for study eligibility. Inclusion and exclusion criteria are listed in Fig. 1. Two senior surgeons (CF and CH) performed all the surgeries in both groups. Preoperatively, all patients had sustained an ACL tear, diagnosed based on clinical examination, MRI and arthroscopically.

MRI examinations were performed using the department protocol with the patient in the supine position and the knee extended (a maximum of 10° of slight flexion of the knee was allowed in case of marked effusion) on a 3-T whole-body scanner (Skyra, Siemens Healthineers, Er- langen, Germany) using a 6-channel flex coil. MRI was performed within 3 weeks from trauma. Three plane (sagittal, coronal, and axial) sequences using both proton density– and fat-suppressed proton density–weighted images were performed with repetition time (TR) between 3000 and 4000 ms, echo time (TE) between 33 and 35 ms, matrix between 320 × 320 and 384 × 384 (phase x frequency) with 3-mm slice thickness, and a total field of view of 130 mm.

### Axial femorotibial alignment

**Fig. 2:**
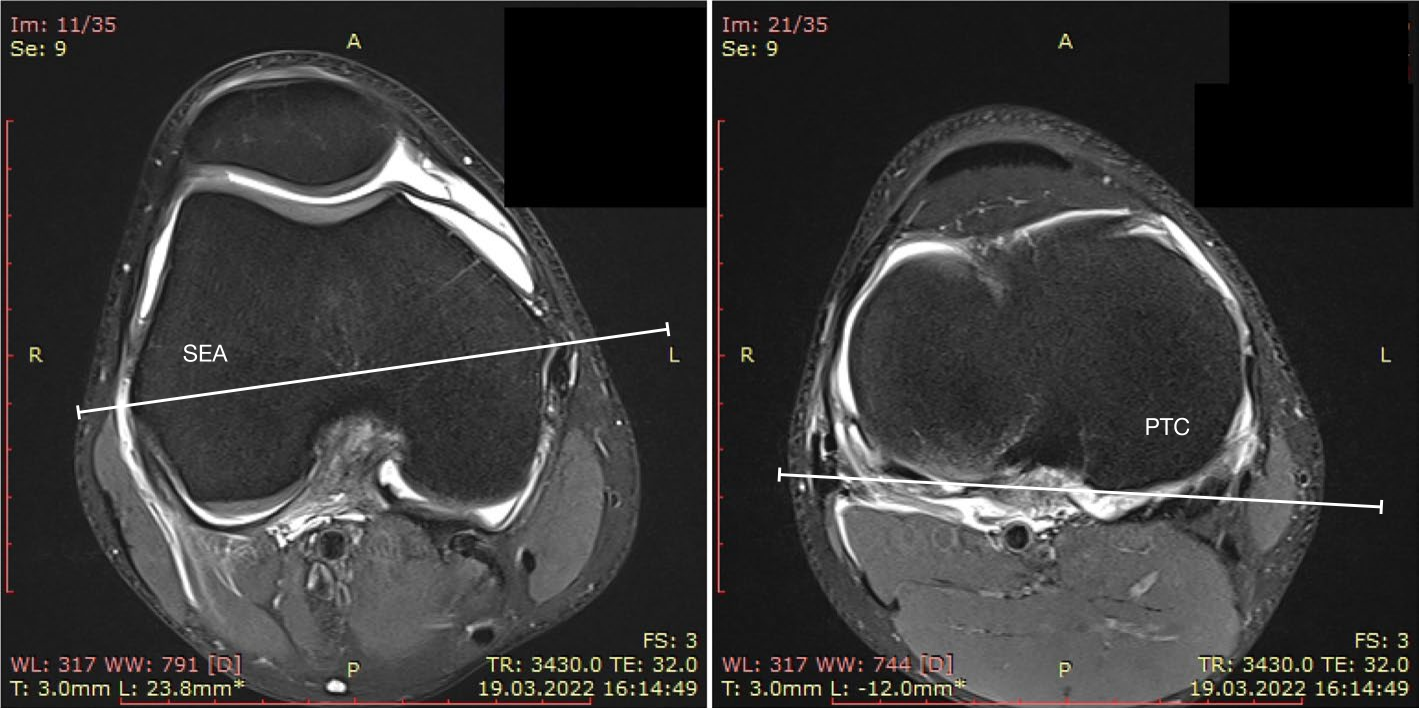
SEA – PTC angle

Two sports knee surgery fellows (LF and AM) independently analyzed all MRI images obtained before surgery. To measure the axial alignment of the distal femur and proximal tibia, two sections were identified from each MRI as described by previous studies [11, 12]. The first slice was taken in the midthrochlear region of the femoral condyle, identified by the Roman arch appearance of the intercondylar groove with the apex of the Roman arch corresponding to 1/3 of the height of the condyle. The surgical epicondylar axes (SEA) from the lateral epicondyle and medial sulcus were delineated. The second slice was taken in correspondence with the proximal tibial plateau above the end of the proximal tibiofibular joint where the semimembranosus tendon inserts into the tibial bone. The tangent line of the posterior tibial condyles (PTC) was delineated. The angle between SEA-PTC was measured (Fig. 2). A negative value was defined as internal torsion and a positive value as external torsion of the distal segment.

### Kaplan fiber complex identification

**Fig. 3:**
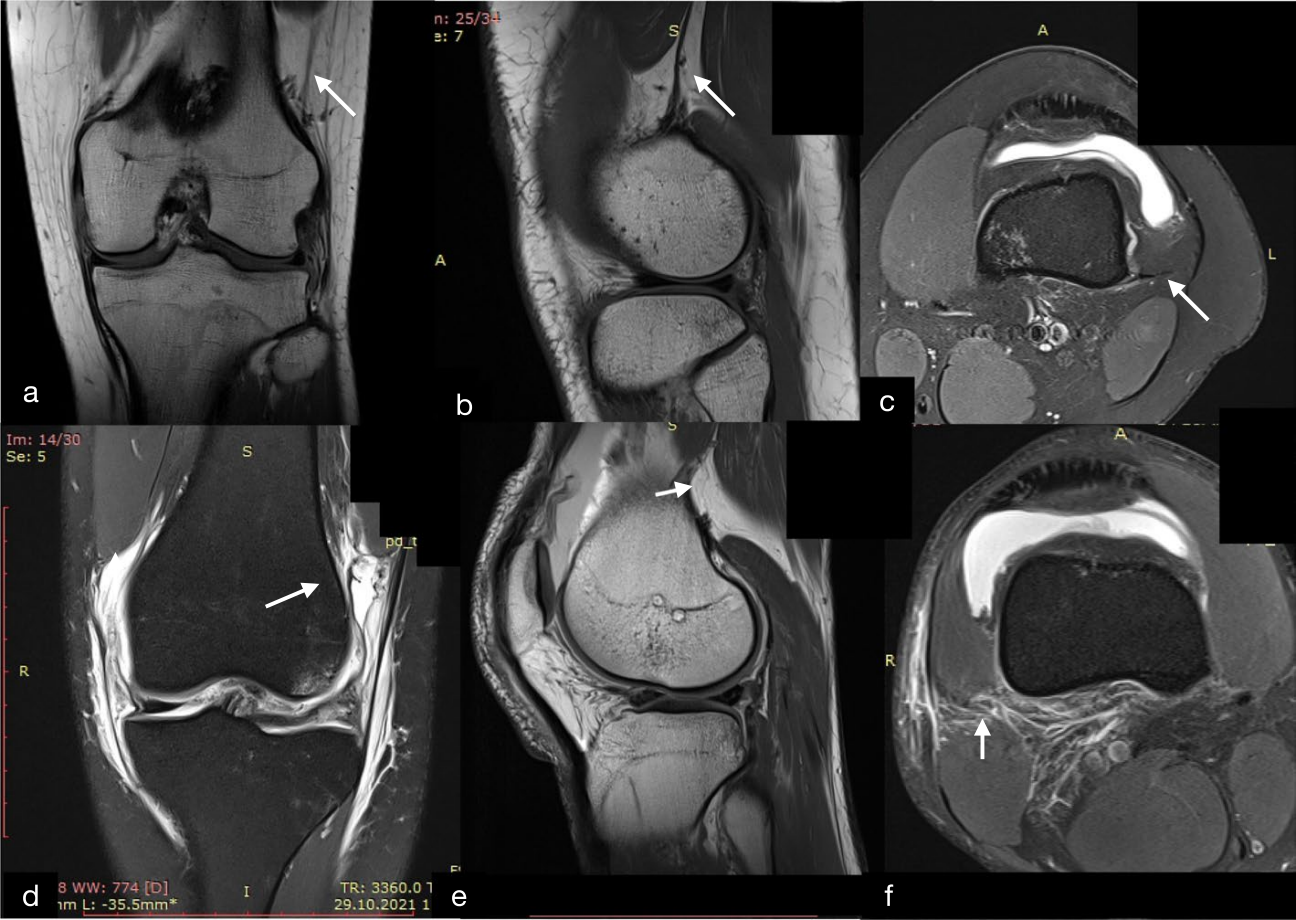
**a** Coronal proton density, **b** Sagittal proton density, and **c** axial fat-suppressed T2 magnetic resonance imaging sections of knee depicting the integrity of distal Kaplan fibers (KFs) complex (white arrow). **d** Coronal fat-suppressed T2, **e** Sagittal proton density, and **f** axial fat-suppressed T2 magnetic resonance imaging sections of knee depicting the injuries of distal Kaplan fibers (KFs) complex (white arrow). The wavy appearance of KFs, discontinuity of KFs and soft tissue oedema in the region of KFs are visualized respectively in (**d**, **e** and **f**)

All scans were reviewed in all 3 planes (axial, coronal, and sagittal) as previously described by studies of Batty et al. [1, 2] and Godin et al. [19]. As a routine, the sagittal images were assessed first with the lateral gastrocnemius origin and superior lateral geniculate artery used as a reference to easily localize the region [37]. Indeed, the KFs were identified proximal to the lateral femoral condyle, adjacent to the branches of the superior lateral genicular artery [3]. Once identified, further assessment using proton density sequences was preferable to visualize KFs with greater clarity. The morphology of the femoral insertion could be different, ranging from a single thick linear insertion to the appearance of multiple smaller strands inserting individually from the posterolateral femur to the epicondylar region. In accordance with Batty et al. [2], KFs were classified as injured if there were: (1) direct signs of injury such as a clear discontinuity in the KFs or a femoral avulsion was visible or (2) there were indirect signs of injury such as thickening and/or intra-substance signal change of the KFs, focal bone marrow oedema at KFs insertion site to the femur, soft tissue oedema in the region of KFs or a wavy appearance to the KFs (Fig. 3).

### Anterolateral ligament identification

**Fig. 4:**
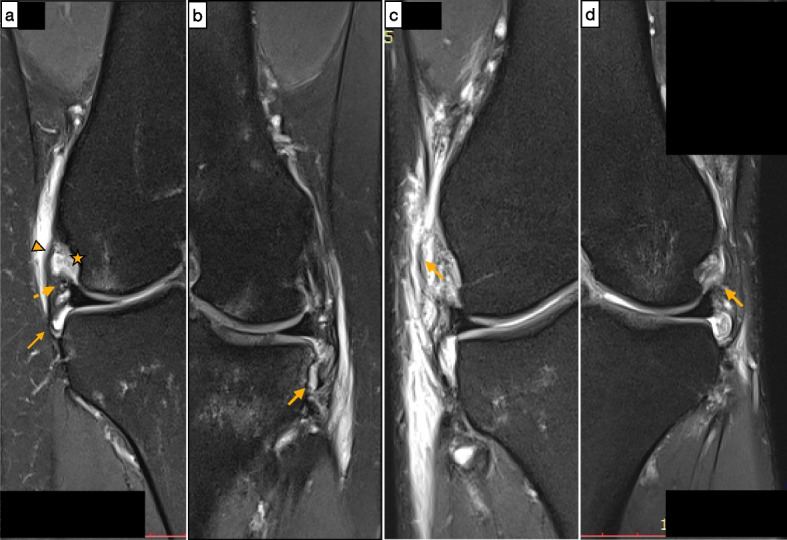
**a** Coronal T2-weighted images with fat saturation shows the normal femoral portion (arrowhead), meniscal portion (dotted arrow) and tibial portion (arrow) of anterolateral ligament; popliteus tendon (star);** b** Segond avulsed bone fragment (arrow); **c** femoral portion of ALL presenting abnormal signal and irregular aspect of its fibers (arrow); **d** meniscal portion of ALL presenting abnormal signal and irregular aspect of its fibers (arrow)

All scans were reviewed in all 3 planes (axial, coronal, and sagittal) as previously described by Helito et al. [20–23]. The ALL was evaluated by the use of T2-weighted coronal images, with the axial and sagittal planes used mainly for anatomic orientation [15, 34]. The ALL was defined as the low signal band originating from the posterior-proximal region of the lateral epicondyle of the femur to its tibial insertion between the Gerdy’s tubercle and the fibular head [44]. Specifically, the ALL was divided into three parts (femoral, meniscal and tibial portion) based on previous anatomic studies (Fig. 4a) [20, 21]. The fibers were considered injured in case of Segond fracture or when they presented irregular contours, a wavy aspect, or areas of discontinuity [15, 25]. The ALL was defined as injured if at least one of its portions resulted in tearing (Fig. 4).

### Statistical analysis

Data were collected and analyzed using respectively Excel (Microsoft, Redmond, WA, USA) and XLSTAT statistical software packages (Addinsoft LLC, Paris, France). Categorical variables were expressed in numbers and percentages (%). The distribution of continuous variables was assessed by Shapiro–Wilk test. Mean and standard deviation (SD) or Median and interquartile ranges (IQR) were used to summarize the continuous variables according to their distribution. A 2-way random interclass correlation coefficient (ICC) was used to assess the interobserver reliability of the KFs and ALL injuries. ICC values were calculated for absolute agreement and consistency of agreement. ICC values were graded as follows: < 0.4 poor reliability, 0.4–0.75 moderate reliability, and > 0.75 excellent reliability [32]. An *F-test* was used to determine the statistical significance of the correlations. Analysis of covariance (ANCOVA) was performed to examine the independent associations between SEA-PTC angle and explanatory variables as ALL and KFs injuries adjusted for physical variables (age, gender and body mass index [BMI]). Mann–Whitney or Kruskall-Wallis test was used to assess significance between groups of continuous variables. Statistical significance was set at a *p-value* of < 0.05.

## Results

**Fig. 5:**
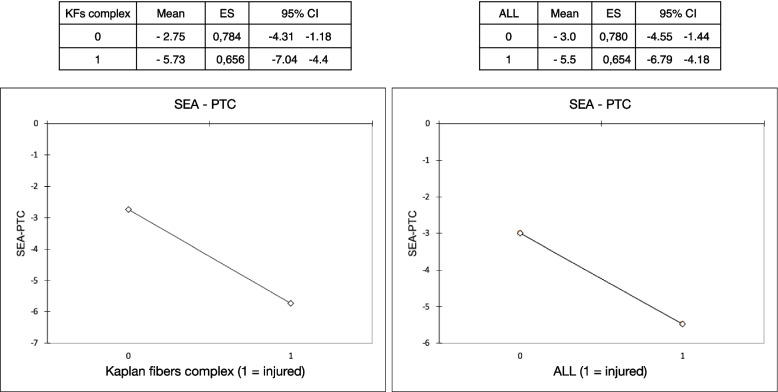
Estimated SEA-PTC angle in relation to depending variable Kaplan fiber complex injuries (KFs) and ALL injuries. SE: standard error; CI: confidence interval

**Fig. 6:**
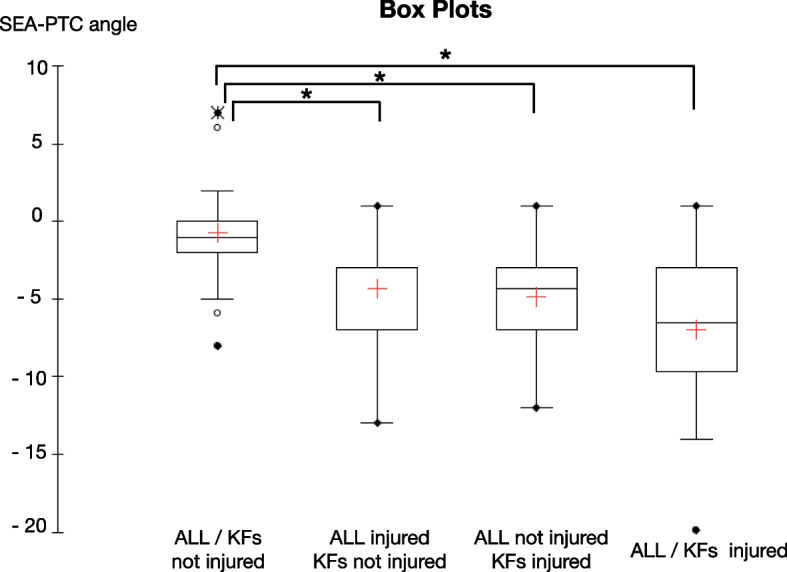
The value of SEA-PTC angle in relation of ALL and KFs injuries. + : mean; * significant with *p* < .05; ALL: anterolateral ligament; KFs: Kaplan fibers complex

The flowchart of the study is presented in Fig. 1. A total of 83 eligible patients were included in the study. The mean age of patients was 24.8 (range 18–53) and 41 were males (49.3%) (Table 1). Interrater reliability analysis revealed an ICC of 0.802 (95% CI 0.711 – 0.864), *p* < .0001 and 0.857 (95% CI 0.778—0.908), *p* < .0001 respectively for KFs and ALL injuries. The prevalence of ALL and KFs injuries in our cohort was 59.0% (Table 2). The result of multiple linear regression analysis adjusted for physical variables showed that internal tibial rotation was associated with KFs and ALL injuries with an odds ratio respectively of 1.36 (95% CI 1.10 – 1.67, *p* = 0.005) and 1.29 (95% CI 1.05 – 1.59, *p* = 0.017) (Table 3). Hence, the estimated average of SEA-PTC angle in relation to ALL injuries controlling the other variables was -5.49 [95%CI -6.79 – (-4.18)] versus -2.99 [95%CI -4.55 – (-1.44)] without ALL injuries (Fig. 5). On the other hand, the estimated average of SEA-PTC angle in relation to KFs lesions controlling the other variables was -5.73 [95%CI -7.04 – (-4.43)] versus -2.75 [95%CI -4.31 – (-1.18)] without KFs injuries (Fig. 5). Furthermore, the value of SEA-PTC angle variable was analyzed considering ALL and KFs injuries (Table 4). Hence, four groups were constituted: ALL / KFs injured (*n* = 32); ALL injured / KFs not injured (*n* = 17); ALL not injured / KFs injured (*n* = 17) and ALL / KFs not injured (*n* = 17) (Table 4). The mean of SEA-PTC differs significantly between groups (*p* < 0.001, Kruskal–Wallis test). Specifically, ALL / KFs injured group reported the greatest internal tibial rotation with a SEA-PTC angle that differs significantly from ALL / KFs not injured group (*p* < .001, Mann–Whitney U test). Considering the groups without KFs injuring, the presence of ALL tears was associated with significant greater internal tibial rotation (*p* = .011). Similarly, the presence of KFs tears in groups without ALL injuring, was associated with significant greater internal tibia rotation (*p* = .004) (Fig. 6).

**Table 1:** Patient’s demographic characteristics

*Variables*	
Age, years	
Mean (SD, range)	24.8 (6.8, 18—53)
Median (IQR, 1^st^ – 3^rd^ quartile)	24 (8, 20—28)
Gender, Male N, %	41 (49.3%)
Tegner activity scale score	
Mean (SD, range)	6.8 (1.3, 3 – 10)
Median (IQR, 1^st^ – 3^rd^ quartile)	7 (1, 6 – 7)
Body-mass-index (BMI), Kg/m^2^	
Mean (SD, range)	23.1 (2.5, 18.9 – 34.0)
Median (IQR, 1^st^ – 3^rd^ quartile)	23.1 (2.7, 21.5 – 24.2)

*SD* Standard deviation, *IQR* Interquartile range

**Table 2:** Anatomic characteristics of the population

*Variables*	
SEA-PTC angle** °**	
Mean (SD, range)	-4.7 (4.9, -19.9 – 7)
Median (IQR, 1^st^ – 3^rd^ quartile)	-3.9 (7.0, -8.0 – (-1))
Kaplan Fibers complex injuries, N (%)	49 (59.0%)
ALL injuries, N (%)	49 (59.0%)

*SD* Standard deviation, *IQR* Interquartile range

**Table 3:** Analysis of covariance (ANCOVA) shows the association between SEA-PTC angle and Kaplan fiber complex (KFs) and anterolateral ligament (ALL) injuries adjusted for physical variables

Factors	Regression coefficient (β)	SE	OR (95% CI)	*P* value
Age	-0.104	0.105	0.90 (0.73 – 1.11)	0.326
BMI	0.103	0.109	1.11 (0.89 – 1.38)	0.349
Gender	0.050	0.107	1.05 (0.85 – 1.30)	0.640
ALL injuries	0.253	0.104	1.29 (1.05 – 1.59)	**0.017**
KFs injuries	0.304	0.105	1.36 (1.10 – 1.67)	**0.005**

*BMI* Body mass index; *R*^2^ = 0.22

**Table 4:** The value of SEA-PTC angle in relation of KFs and ALL injuries

	ALL Injured	ALL not injured	*P* value*
KFs complex injured	-6.9 (5.2)	-4.9 (3.6)	.219
KFs complex not injured	-4.4 (3.8)	-0.8 (3.7)	**.011**
.532	.073	**.004**	** < .001**

Data are reported by mean and standard deviation (SD)

* Significance has been assessed by Mann–Whitney U test

## Discussion

The most important finding of the present study was that KFs and ALL injuries were associated with an increased intra-articular internal tibia rotation in ACL-deficient knees based on high-field MRI. The ALC has been described as including the iliotibial band (ITB), the KFs complex, the capsule-osseous layers of ITB, the anterolateral capsule and ALL even though debating and conflicting results are present in literature due to the complexity of lateral knee anatomy and various dissecting techniques [39, 40]. Biomechanical studies have shown that the ALC has a role as secondary stabilizer to the ACL in opposing anterior tibial translation and internal tibial rotation [27, 31, 39, 41–43, 46, 48]. Moreover, additional investigations demonstrated that ACL reconstruction alone in the setting of combined ACL and ALC injuries failed to restore native knee kinematics [28, 31].

Previous studies by Van Dyck et al. [49] and Khanna et al. [30] reported respectively a rate of KFs complex injuries of 33% and 82%, whereas Batty et al. [2] reported a lower rate of KF injuries (18.6%) in patients with ACL-deficient knees. In the former studies the determination of injury was based on the presence of any altered signal within the ligament, periligamentous oedema and/or disruption of the fibers whereas in the latter probably more restrictive criteria were used [2]. From a biomechanical point of view, KFs complex has been described as an important factor controlling anterolateral rotatory stability [17, 31, 35]. Specifically, injuries to KFs complex concomitant to ACL deficit were associated to greater internal tibial rotation in laboratory studies [31]. Similarly, from our results a greater internal tibial rotation on MRI was associated to KFs complex injuries with Odds ratio of 1.36 (95%CI 1.10 – 1.67, *p* = .005).

From our cohort, we reported a rate of ALL injuries of 59% in ACL-deficient knee. Claes et al. [7] and Ferretti et al. [14] reported the prevalence of ALL abnormalities in the ACL-injured knee respectively in 80% and 90% of cases, but in others the rate was approximately 50% [5, 23, 24]. Biomechanical studies have shown that ALL injuries were associated with an increase internal rotation of the tibia in ACL-injured knee specially when knee flexion exceeds 35° [41, 43, 45]. In our study, we reported that injuries to KFs complex was associated with an increase internal tibial rotation with an odds ratio of 1.29 (95%CI 1.05 – 1.59, *p* = 0.017).

Considering the magnitude of internal tibial rotation in our cohort, patients with both ALL and KFs complex injuries were characterized by greatest internal tibial rotation compared to other groups assuming a synergistic effect of ALL and KFs complex in controlling anterolateral rotatory knee laxity [42]. In addition, analyzing the SEA-PTC variable in KFs injuries groups, we observed that ALL injuries did not significantly increase the internal rotation of the tibia (*p* = .219). A possible explanation could be that ALL has been demonstrated to act as a secondary stabilizer during internal rotation torque and simulated pivot-shift test in the ACL-deficient state over 30° of flexion whereas in our study the internal rotation was measured nearly in full extension due to MRI examination [44].

On the other hand, analyzing the SEA-PTC variable in KFs not injured groups, we observed that ALL injuries significantly increased the internal tibial rotation (4.4° vs 0.8°, *p* = .011). These results are similar with those of Spencer et al., who reported, after ALL sectioning in an ACL-sectioned knee, a significant increase in internal tibial rotation of only 2° at full knee extension. This amount was assumed by authors to be clinically undetectable and consistent with a secondary restraint to internal rotation [46].

Lateral extra-articular tenodesis (LET) and anterolateral ligament (ALL) reconstructions have been shown to restore knee kinematics in the setting of combined anterolateral instability and ACL injuries [8, 29, 31]. For these reasons, adding ALL reconstruction or LET should be considered in case of ALL and/or KFs complex injuries in order to decrease the chronic residual laxity after isolated ACLR [17, 28].

The present study has limitations that warrant disclosures. First, the ALL and KFs complex injuries were diagnosed on unvalidated diagnostic criteria and not confirmed by surgical exploration. There is undoubtedly variability and an element of subjectivity in evaluating the anterolateral structures on MRI. However, the ICC values showed excellent agreement and the methodology was the same as previous dedicated studies [2, 23, 26]. Only the distal fibers of KFs complex were considered in the present study because the most proximal fibers were outside the MRI field of view. Femoral anteversion, tibial torsion and contralateral femoral tibial rotation of the knee were not considered, representing limitations of our study. All MRIs were performed within 3 weeks from trauma, therefore the rate of KF injuries might be overestimated due to the presence of widespread oedema in acutely injured knees [2]. However, larger quantities of fluid inside the joint puts tension on the capsule and makes it easier to view the ALL compared to chronic cases [21]. The study was carried out by analyzing static measurement (static MRI of knee performed after trauma). For this reason, the different behavior of the various components of the ALC as the flexion angle of the knee increases, cannot be considered. Therefore, dynamic analysis is necessary to confirm the hypothesis. However, Carpenter et al. [4] in a study using three-dimensional MRI showed that knees with ACL reconstruction presented greater internal tibial rotation in going from extension to flexion than those with a native ACL. Authors hypothesized that reconstruction alone did not fully restore the kinematics of the knee maybe due to an undiagnosed and untreated ALC injury.

## Conclusion

ALL and KFs injuries were associated with an increased internal tibial rotation in ACL-deficient knees on high-field MRI. The measurement of femorotibial rotation on axial MRI could be useful to detect indirect signs of ALC injuries which could help in the diagnosis and management of patients with these injuries. Further studies are required to assess and validate the measurement of femorotibial rotation on axial MRI as an indirect measure of rotatory instability in ACL-deficient knees.
